# Posttraumatic Stress after SARS

**DOI:** 10.3201/eid1108.041083

**Published:** 2005-08

**Authors:** Kitty K. Wu, Sumee K. Chan, Tracy M. Ma

**Affiliations:** * Caritas Medical Centre, Hong Kong;; † Princess Margaret Hospital, Hong Kong;; ‡ Kwong Wah and Wong Tai Sin Hospitals, Hong Kong

**Keywords:** trauma, resilience, Impact of Event Scale, Hospital Anxiety and Depression Scale, infectious disease

## Abstract

Posttraumatic stress disorder (PTSD) can arise in patients with medical illness. We used 2 Chinese self-report measures to examine features of PTSD, anxiety, and depression in 131 survivors of severe acute respiratory syndrome at 1 month and 3 months after discharge from the hospital. Risk factors associated with psychological distress were identified.

In the outbreak of severe acute respiratory syndrome (SARS) in Hong Kong in 2003, a total of 1,755 people were infected, and 299 died. Among the infected, 386 were healthcare workers (1). According to the literature, a life-threatening physical illness can lead to symptoms associated with posttraumatic stress disorder (PTSD) after recovery. The prevalence rates of PTSD are 1%–5% for childbirth and 14%–59% for a life-threatening situation in an intensive care unit (ICU) (2). Predictors of PTSD identified in previous studies included aspects of the trauma itself, emotional support, and invasiveness of the medical intervention. Severity of illness was not correlated with the development of PTSD (2).

Previous studies showed that 10%–35% of SARS survivors reported having features of anxiety, depression, or both at 1 month after discharge (3–6). Repeated measures of the effect at different time points beyond 1 month after discharge are needed to understand the psychological sequelae related to SARS and enrich the understanding of the long-term psychological functioning of survivors of life-threatening infectious disease.

## The Study

This study examined the psychological sequelae related to SARS at 1 month and 3 months after discharge from hospital. According to previous studies on posttraumatic stress, 3 categories of risk factors were postulated. The first category included pre-SARS variables: sex, age, education level, family income, availability of emotional support as indicated by the number of persons with whom one could talk and share worries, and whether one was a healthcare worker. The second category included parameters for severity of disease and treatment regimen: lowest level of blood oxygen saturation (SaO_2_) during hospitalization, duration of hospitalization for SARS treatment, whether treatment in ICU was required, and total steroid dosage used during hospitalization. The third category was SARS-related psychological and social variables: whether the participant knew anyone who was suspected or confirmed to have SARS, whether the participant knew anyone who died of SARS, and rating for subjective sense of threat.

The assessment materials printed in Chinese were mailed to 476 SARS patients l month and 3 months after they were discharged from the hospital. Of the 476 SARS survivors contacted, 25 were healthcare workers. One hundred ninety-five (41%) respondents returned the completed questionnaires at 1 month after discharge; characteristics and survey results for the psychological adjustment of these respondents were previously documented (5). A total of 131 (28%) respondents responded at both l month and 3 months after discharge. Our study was based on the data for these 131 respondents. No significant difference was seen between the 3-month respondents and nonrespondents for all the variables examined at 1 month.

Among the 131 participants, ages were 18–84 years (mean 41.82, standard deviation [SD] 14.01); 57 (44%) were men, 74 (56%) were women. Fourteen (11%) were healthcare workers, 4 (3%) had a history of psychiatric consultation, 12 (9%) had other chronic diseases, and 15 (11%) required treatment in the ICU. The lowest level of SaO_2_ during hospitalization was 79%–96% (mean 91.59%, SD 3.29). The total steroid dosage used for treatment ranged from 0 to 86,900 mg (mean 14,120.28 mg, SD 12,254.95).

Fifty-seven participants (44%) personally knew someone who was suspected or confirmed to have SARS. Fourteen (11%) knew someone who died of SARS. Regarding the number of persons with whom they could talk and share their worries, 6 participants (5%) indicated no one, 68 (52%) indicated 1–2, 41 (31%) indicated 3–4, and 16 (12%) indicated ≥5. For the rating on subjective sense of threat caused by the disease, 11 participants (8%) reported "not at all," 37 (28%) reported "a little," 43 (33%) reported "moderate," 28 (21%) reported "quite serious," and 12 (9%) reported "extremely serious."

The measures used in the study include the Chinese versions of the Impact of Event Scale – Revised (IES-R) (7,8) and the Hospital Anxiety and Depression Scale (HADS) (8–10). Based on research on PTSD and the Diagnostic and Statistical Manual of Mental Disorders, Fourth Edition (DSM-IV) (11), intrusion, avoidance, and hyperarousal were identified as the primary domains of measurement on the IES-R. Scoring was based on previous studies that indicated a mean subscale score of 2, representing a moderate level of distress, is the appropriate cutoff point (7,8).

The Chinese HADS (8–10) is a self-report instrument designed to detect symptoms related to anxiety and depression. As in previous studies, the marker for a moderate level of distress (i.e., the subscale score of 11) was used as the cutoff point for HADS subscale scores (8–10).

Repeated measures analysis of variance (ANOVA), with time (1 month vs. 3 months after discharge) as the within-subject factor, was used to examine the change of symptom severity. The 1-month scores for IES-R intrusion (mean 1.12, SD 0.73), hyperarousal (mean 1.05, SD 0.79), and HADS anxiety (mean 5.87, SD 3.89) were significantly higher than the 3-month scores for IES-R intrusion (mean 0.91, SD 0.73), F_1, 130_ = 18.52, p<0.001), hyperarousal (mean = 0.85, SD = 0.74), F_1, 130_ = 13.96, p<0.001, and HADS anxiety (mean 5.19, SD 4.48), F _1, 130_ = 5.23, p<0.05).

At 1 month after discharge, the number of participants who surpassed the subscale cutoff was 16 (12%) for intrusion, 12 (9%) for avoidance, 19 (15%) for hyperarousal, 17 (13%) for anxiety, and 23 (18%) for depression. At 3 months after discharge, 13 participants (10%) surpassed the subscale cut-off for intrusion, 11 (8%) for avoidance, 12 (9%) for hyperarousal, 18 (14%) for anxiety, and 17 (13%) for depression. As PTSD is characterized by the presence of all 3 symptom clusters (i.e., intrusion, avoidance, and hyperarousal), the percentage of participants who passed the cutoff for all IES-R subscales was examined. Among the 131 participants, 6 (4%) at 1 month and 7 (5%) at 3 months postdischarge had all 3 IES-R subscale scores above the cutoffs.

Results of multivariate analysis of variance indicated significant difference in the combined dependent variables between participants with and without a history of psychiatric consultation (Wilks = 0.83, F test_5, 125_ = 5.18, p<0.001, effect size 0.17). Results in univariate F tests suggested that having a history of psychiatric consultation, being a healthcare worker, and knowing someone who had SARS were associated with various IES-R and HADS scores (Table 1).

**Table 1:** Results obtained for predictors with significant group difference in IES-R and HADS subscales (N = 131)*

	IES-R intrusion		IES-R avoidance		IES-R hyperarousal		HADS anxiety		HADS depression	
	M (SD)	F	M (SD)	F	M (SD)	F	M (SD)	F	M (SD)	F
History of psychiatric consultation										
Yes (n = 4)	2.31 (0.80)	17.24§	1.71 (0.084)	5.73‡	2.33 (0.80)	18.70§	11.50 (4.12)	8.61‡	9.50 (5.25)	4.21†
No (n = 127)	0.86 (0.68)		0.84 (0.70)		0.80 (0.68)		5.00 (4.36)		5.03 (4.25)	
Healthcare worker										
Yes (n = 4)	1.28 (0.89)	4.26†	1.16 (0.73)	2.43	1.35 (0.77)	7.61‡	7.14 (4.94)	2.99	6.85 (4.12)	2.39
No (n = 127)	0.86 (0.69)		0.84 (0.72)		0.79 (0.71)		4.96 (4.39)		4.97 (4.33)	
Know someone to have SARS										
Yes (n = 4)	1.01 (0.66)	2.09	1.00 (0.75)	2.98	0.96 (0.75)	2.42	6.07 (3.93)	3.89	6.12 (4.24)	4.96†
No (n = 127)	0.83 (0.76)		0.77 (0.70)		0.76 (0.72)		4.52 (4.78)		4.44 (4.28)	
*IES-R, Impact of Event Scale – Revised; HADS, Hospital Anxiety and Depression Scale. †p<0.05. ‡p<0.01. §p<0.001.										

Results of Pearson correlations (Table 2) showed that the level of SaO_2_, the number of persons with whom one could talk and share worries, and the rating on perceived threat were significantly related to various IES-R and HADS subscale scores. To determine which variables had the greatest effect on symptom severity at 3 months after discharge, standard multiple regressions were conducted with IES-R and HADS subscale scores as the criterion variables. The lowest level of SaO_2_ during hospitalization, the rating for subjective sense of threat, and the number of persons with whom one could talk and share worries were entered into the regression model as predictor variables. Overall, the amount of total variance accounted for in individual IES-R and HADS subscale scores by these variables was significant. The lowest level of SaO_2_ during hospitalization was the most significant predictor for intrusion and avoidance scores. High level of perceived threat was the most significant predictor for hyperarousal and the HADS anxiety scores. The number of persons with whom one could talk was the most important predictor for the HADS depression score.

**Table 2:** Summary of multiple regression analyses of IES-R and HADS subscales (N = 131)*

Predictor variables	B	ß	sr^2^	R	R^2^	Overall F
Regression analysis to predict IES–R intrusion score						
Subjective threat	0.12	0.18	0.03†	0.22‡		
Emotional support	–0.17	–0.18	0.03†	–0.21‡		
SaO_2_	–0.04	–0.22	0.04†	–0.26‡	0.13	F_3, 127_ = 6.57§
Regression analysis to predict IES–R hyperarousal score						
Subjective threat	0.13	0.19	0.04†	0.22‡		
Emotional support	–0.14	–0.15	0.02	–0.17†		
SaO_2_	–0.02	–0.13	0.02	–0.16†	0.09	F_3, 127_ = 4.15†
Regression analysis to predict IES–R avoidance score						
Subjective threat	0.15	0.22	0.05‡	0.26‡		
Emotional support	–0.17	–0.19	0.03†	–0.22‡		
SaO_2_	–0.05	–0.24	0.06‡	–0.29§	0.17	F_3, 127_ = 8.62§
Regression analysis to predict HADS anxiety score						
Subjective threat	0.73	0.18	0.03†	0.20‡		
Emotional support	–0.77	–0.13	0.02	–0.15†		
SaO_2_	–0.16	–0.12	0.01	–0.15†	0.07	F_3, 127_ = 3.43*
Regression analysis to predict HADS depression score						
Subjective threat	0.24	0.06	0.00	0.09		
Emotional support	–10.03	–0.18	0.03†	–0.20†		
SaO_2_	–0.18	–0.14	0.02	–0.16†	0.06	F_3, 127_ = 20.91†

*IES-R, Impact of Event Scale – Revised; HADS, Hospital Anxiety and Depression Scale, Subjective threat, subjective sense of threat; emotional support, number of persons one could talk to and share worries with; SaO_2_, lowest level of blood oxygen saturation during hospitalization. †p<0.05. ‡p<0.01. §p<0.001.

## Conclusions

The occurrence rate of PTSD features for SARS survivors is in the middle of the range reported in previous samples of other medical diseases (2). For most SARS survivors, a significant decrease in symptom severity from l month to 3 months after discharge was identified.

The significant predictive value of SaO_2_ as an index of disease severity in this study suggests that direct physiologic measures may be more sensitive as indexes of disease severity than other indexes, which could be confounded by other factors (e.g., treatment regimen). Our findings imply that mobilization of resources for emotional support may enhance resilience of SARS survivors. SARS survivors who were healthcare workers, knew someone who had SARS, or had a history of psychiatric consultation had a higher risk for psychological distress and may warrant early and focused support services.

Our study was limited by the low response rate and small sample size of certain groups of participants (e.g., healthcare workers and patients with history of psychiatric consultation). As criterion A in DSM-IV for PTSD, which focuses on the nature and personal response involved in the traumatic experience, was not specifically assessed, the occurrence rate of PTSD could not be taken as a prevalence estimate for PTSD in a straightforward manner. Such findings could better be substantiated by clinical interviews. The study is limited by the availability of a comparison group. Since SARS is a new disease, the psychological effect of some possible long-term physical outcomes related to the disease and treatment regimen (e.g., avascular necrosis) that were not discovered until recently were not captured.
